# The therapeutic potential of bone marrow‐derived macrophages in neurological diseases

**DOI:** 10.1111/cns.13964

**Published:** 2022-09-06

**Authors:** Kai Zhou, Jinming Han, Yafeng Wang, Yiran Xu, Yaodong Zhang, Changlian Zhu

**Affiliations:** ^1^ Henan Neurodevelopment Engineering Research Center for Children Children's Hospital Affiliated to Zhengzhou University Zhengzhou China; ^2^ Department of Neurology, Xuanwu Hospital Capital Medical University Beijing China; ^3^ Department of Hematology and Oncology Children's Hospital Affiliated to Zhengzhou University, Henan, Children's Hospital, Zhengzhou Children's Hospital Zhengzhou China; ^4^ Henan Key Laboratory of Child Brain Injury and Henan Pediatric Clinical Research Center The Third Affiliated Hospital and Institute of Neuroscience, Zhengzhou University Zhengzhou China; ^5^ Centre for Brain Repair and Rehabilitation Institute of Neuroscience and Physiology, Sahlgrenska Academy, University of Gothenburg Gothenburg Sweden

**Keywords:** bone marrow transplantation, dysfunctional microglia, gene delivery, gene therapy, macrophage, monocyte

## Abstract

Circulating monocytes are precursors of both tissue macrophages and dendritic cells, and they can infiltrate the central nervous system (CNS) where they transform into bone marrow‐derived macrophages (BMDMs). BMDMs play essential roles in various CNS diseases, thus modulating BMDMs might be a way to treat these disorders because there are currently no efficient therapeutic methods available for most of these neurological diseases. Moreover, BMDMs can serve as promising gene delivery vehicles following bone marrow transplantation for otherwise incurable genetic CNS diseases. Understanding the distinct roles that BMDMs play in CNS diseases and their potential as gene delivery vehicles may provide new insights and opportunities for using BMDMs as therapeutic targets or delivery vehicles. This review attempts to comprehensively summarize the neurological diseases that might be treated by modulating BMDMs or by delivering gene therapies via BMDMs after bone marrow transplantation.

## Graphical abstract

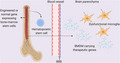

## 1 INTRODUCTION

Monocytes differentiate from monoblasts in the bone marrow before moving into the bloodstream, and they account for 2%–8% of the total leukocytes in the blood.^1^ Over 50% of monocytes are reserved in the spleen and accumulate rapidly after injury.^2^ Three types of circulating monocytes in humans have been identified based on the expression of CD14 and CD16 and are classified as CD14^high^/CD16^−^, CD14^low^/CD16^high^, and CD14^high^/CD16^low^ monocytes.^3^ In addition, two subsets of circulating monocytes have been identified in mice, namely CX3CR1^high^/CCR2^−^/Ly6C^low^/PD‐L1^+^ and CX3CR1^low^/CCR2^+^/Ly6C^high^/PD‐L1^−^ monocytes, and they can be recruited to both inflamed and noninflamed sites or can patrol along the blood vessels, respectively.^1^, ^4^, ^5^ Moreover, monocytes can differentiate into tissue macrophages or dendritic cells after infiltrating into the tissue.^5^, ^6^

Circulating monocytes cannot infiltrate into the brain under physiological conditions due to the blood–brain barrier (BBB). However, CX3CR1^low^/CCR2^+^/Ly6C^high^/PD‐L1^−^ monocytes can penetrate the brain and become bone marrow‐derived macrophages (BMDMs) under some disease conditions in which the BBB is compromised.^7^, ^8^, ^9^, ^10^ However, the cellular and molecular mechanisms regulating monocyte infiltration are largely unknown. Cell adhesion molecules such as intercellular adhesion molecule‐1 and vascular cell adhesion molecule‐1 expressed in endothelial cells have been shown to play critical roles in monocyte infiltration, and these molecules have been used to regulate monocyte infiltration and neuroinflammation.^11^, ^12^, ^13^ Interestingly, previous studies have suggested that the process of monocyte infiltration does not require BBB damage. Instead, C‐C motif chemokine receptor 2 (CCR2) was noted to be the entry ticket for monocyte infiltration into the CNS. Thus, CCR2 is widely used as a therapeutic target for inhibiting monocyte infiltration into the brain.^7^, ^9^, ^14^, ^15^

Similar to the resident microglia, BMDMs express cellular markers, such as CD11b, Iba‐1, and CX3CR1. However, BMDMs and microglia display distinct functions in disease conditions.^7^, ^16^ Moreover, chimeric mouse models, bone marrow transplantation (BMT), single cell sequencing, and the discovery of new microglial‐specific markers have made it possible to distinguish BMDMs and microglia.^17^, ^18^, ^19^, ^20^, ^21^ BMDMs play paradoxical roles in CNS pathology and recovery,^22^, ^23^ and here we summarize the complex roles that BMDMs play in various CNS diseases and discuss how BMDMs might serve as a potential therapeutic target for treating such disorders.

## 2 BMDMS PLAY COMPLEX ROLES IN VARIOUS CNS DISEASES

**FIGURE 1:**
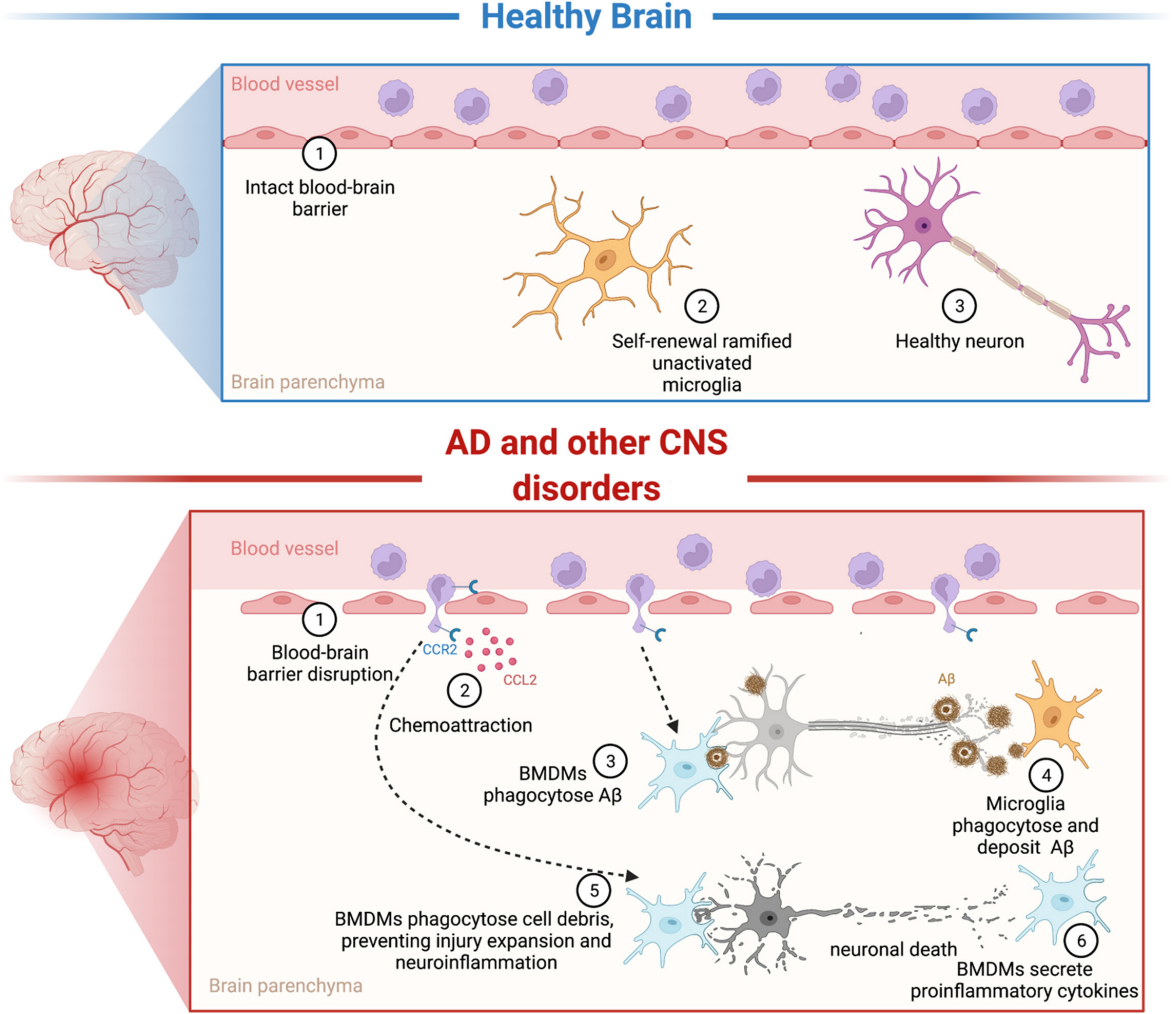
Monocytes in the healthy brain and in AD and other CNS disorders. The top panel illustrates how monocytes circulate in the blood vessels of the healthy brain, with no infiltration into the brain parenchyma. The bottom panel shows how circulating monocytes infiltrate into the brain parenchyma due to BBB disruption and chemoattraction in AD and other CNS disorders.

BMDMs can promote neuroinflammation and exacerbate neurodegeneration in various CNS diseases, but they can also compensate for dysfunctional microglia and can remove toxic substances and cellular debris, thus protecting the brain from further injury (Figure 1). These dual roles of BMDMs have been reported in various CNS disease conditions as described below.

### 2.1 Viral encephalitis

Acute viral infection and the following chronic inflammatory responses cause various behavioral deficits, including motor, psychiatric, and cognitive dysfunctions.^24^ In addition, the infiltration of monocytes is a hallmark of viral infections in the CNS.^25^

Inhibiting monocyte infiltration using CCR2 knockout (KO) mice increased the mortality and duration of West Nile virus (WNV) encephalitis, indicating a beneficial role of BMDMs in treating this disease.^26^ In support of this, the depletion of monocytes using clodronate‐loaded liposomes increased the mortality in a low virus dose‐induced WNV model, which might be due to the loss of phagocytosis of the virus particles.^27^ In contrast, inhibiting monocyte infiltration by blocking adhesion molecule binding or by using an antibody against C‐C motif chemokine ligand 2 (CCL2) revealed a pathologic role for BMDMs in a lethal WNV model, which might be due to the proinflammatory response of BMDMs.^28^, ^29^ Moreover, CCR2 KO decreased hippocampal neuronal death by decreasing BMDM‐derived proinflammatory cytokines in a Theiler's virus model.^30^ Thus, BMDMs appear to play contrasting roles in viral encephalitis, which might be due to the different viral encephalitis models that have been studied and to the different ways of inhibiting the infiltration of monocytes.

### 2.2 Multiple sclerosis (MS)

In an experimental autoimmune encephalomyelitis (EAE) mouse model, electron microscopy showed that BMDMs were attached to nodes of Ranvier and initiated demyelination in the brain.^31^ Moreover, BMDMs were found to secrete large amounts of proinflammatory cytokines and to participate in the inflammatory response in the EAE model.^32^ A strong correlation between BMDMs and the paralytic stage of EAE was also noted.^33^ Furthermore, decreasing monocyte infiltration by blocking endothelial adhesion molecules exerted antiinflammatory effects and reduced the pathological process of MS,^13^, ^34^ and CCR2 KO mice failed to develop pathological lesions in the CNS, indicating the indispensable role of BMDMs in EAE pathology.^35^, ^36^ Conversely, a clinical study suggested that foamy macrophages consisting of both microglia and BMDMs are antiinflammatory in patients with MS.^37^ However, that study lacked evidence to show that they could accurately distinguish between microglia and BMDMs. Taken together, consistent results support the detrimental roles of BMDMs in animal models of MS. However, caution should be used when interpreting the clinical results because of the limitations of the methods for distinguishing microglia from BMDMs.

### 2.3 Traumatic brain injury (TBI)

TBI comprises primary and secondary injury with disruption of the BBB. After the primary injury, circulating monocytes are recruited to the injury site where they might secrete proinflammatory cytokines, reactive oxygen species, and proapoptotic proteins that aggravate the neuronal damage.^38^, ^39^ Moreover, CCR2 KO and CCR2 selective antagonists diminish the TBI‐induced brain injury and cognitive dysfunctions.^40^, ^41^, ^42^, ^43^ Similar results were obtained in the C‐C motif chemokine ligand 2 (CCL2) KO and liposome‐encapsulated clodronate‐induced monocyte depletion model.^44^, ^45^ Furthermore, infiltrating monocytes are more robust in aged TBI mice compared with young TBI mice, and CCR2 KO prevents chronic injury‐induced cognitive decline in the aged TBI mice.^46^ Together, these results indicate that BMDMs play pathological roles in TBI, and therefore, inhibiting the infiltration of monocytes might be an effective strategy to alleviate brain injury.

### 2.4 Spinal cord injury (SCI)

SCI usually causes irreversible disability because of the permanent loss of neurons and the inability for them to regrow. Circulating monocytes infiltrate the injury site after SCI, and BMDMs may help spinal cord recovery after SCI through the expression of antiinflammatory cytokine IL‐10. Moreover, eliminating BMDMs by antigen‐mediated depletion or by exposure to diphtheria toxin reduced the physical recovery after SCI.^47^ Other studies also demonstrated a protective role for BMDMs by resolving neuroinflammation and removing myeline or cellular debris in various animal models of SCI.^48^, ^49^, ^50^, ^51^ Conversely, BMDMs are necessary for forming the fibrotic scar that inhibits axon regeneration.^52^ Moreover, monocyte depletion by liposome‐encapsulated clodronate alleviates SCI‐induced behavioral deficits.^53^, ^54^, ^55^ Taken together, these findings indicate the complex roles of BMDMs in SCI.

### 2.5 Alzheimer's disease (AD)

Amyloid‐β (Aβ) plays a central role in the pathophysiology of AD, and accumulation of Aβ in the extracellular space can be neurotoxic and further induce tau pathology, leading to neurodegeneration. Therefore, targeting Aβ has been used as a therapeutic strategy for early stage AD.^56^ Previous studies revealed that microglia are responsible for Aβ deposition,^57^, ^58^, ^59^, ^60^ and a recent study demonstrated that microglia are Aβ carriers and can transport Aβ to healthy brain tissues.^61^ Conversely, microglia can also degrade and remove Aβ^62^, ^63^ (Figure 1).

Similarly, monocytes play dual roles in the progression of AD.^64^ However, BMDMs have been shown to play a beneficial role in early AD pathology, and one study demonstrated that BMDMs, rather than microglia, can eliminate Aβ by phagocytosis^65^ (Figure 1). Consistent with this, inhibiting the infiltration of monocytes (CCR2 KO) aggravates the progression of AD.^66^, ^67^ Moreover, transplantation of wild‐type bone marrow cells expressing CCR2 can decrease Aβ accumulation and prevent cognitive decline in the CCR2 KO AD mouse model.^68^ All of these findings indicate that BMDMs play neuroprotective roles in controlling the progression of AD, and BMDMs are thus therapeutic targets for AD.^69^ Several approaches targeting BMDMs have shown positive therapeutic effects. For example, systematic administration of macrophage colony‐stimulating factor increased the number of BMDMs in the brain, thus decreasing the Aβ deposition and further rescuing social deficits and cognitive decline.^70^ Taken together, these findings suggest that promoting BMDMs can be an effective treatment for the early stages of AD.

### 2.6 Ischemic stroke

Ischemic stroke accounts for the majority of stroke cases, and the BBB can be severely compromised after ischemic stroke. This leads to the infiltration of circulating monocytes to the injury sites, and BMDMs can remove cellular debris,^71^ supporting angiogenesis,^72^ and resolve ongoing neuroinflammation.^73^, ^74^, ^75^ Moreover, impaired monocyte infiltration due to CCR2 KO or to treatment with CCR2 antagonists can impair angiogenesis, increase the extent of tissue damage, and exacerbate behavioral deficits.^74^, ^75^, ^76^ Furthermore, macrophage colony‐stimulating factor mobilizes circulating monocytes, increases the infiltration of BMDMs into the injury sites, and protects the brain from secondary injury.^77^ Interestingly, both Ly6C^high^ and Ly6C^low^ monocytes in mice can infiltrate into the brain after ischemic stroke and play distinctly protective roles in disease recovery.^78^, ^79^ Notably, one study demonstrated that BMDMs play distinct roles in different stages of ischemic stroke. In the acute phase, BMDMs are proinflammatory and can worsen the brain injury, and CCR2‐deficiency decreases acute injury. However, at later phases, BMDMs gradually switch to antiinflammatory states and are essential for brain recovery, and CCR2‐deficient mice show increased mortality rates and increased delayed neurological injury.^80^ Moreover, modulating BMDMs state from proinflammatory to antiinflammatory can promote brain recovery.^81^, ^82^ Because BMDMs play distinct roles in different phases of ischemic stroke, different intervention strategies may need to be applied at different phases.

### 2.7 Retinal degeneration

Monocytes were shown to infiltrate into the retina, proliferate, and become BMDMs in an N‐methyl‐N‐nitrosourea‐induced retinal damage mouse model.^83^ BMDMs made up 15% of the total microglia 7 days after the injection of N‐methyl‐N‐nitrosourea, and they were explicitly located in the injury sites, which suggests a role in the phagocytosis of cell debris and in resolving inflammation. In an inherited retinal degeneration mouse model, 80% of the microglia were replaced by BMDMs, and decreasing the recruitment of BMDMs into the degenerating retina by inhibiting stromal‐derived factor 1 or by systemic depletion of circulating monocytes resulted in the acceleration of retinal degeneration. Moreover, systemic administrations of granulocyte colony‐stimulating factor 1 and erythropoietin have been shown to slow down retinal degeneration by synergistically stimulating bone marrow stem cells and circulating monocytes.^84^, ^85^

A study demonstrated that BMDMs are neuroprotective in a mouse model of glutamate toxicity in the eye.^86^ The authors showed that the existence of BMDMs in the retina can protect retinal ganglion cells from retinal insult, and they showed the antiinflammatory action of BMDMs by regulating the accumulation of other immune cells. Finally, the authors provided evidence that BMDMs support progenitor cell renewal after retinal injury. Moreover, BMDMs can promote the clearing o of Aβ from the retina, prevent neuron loss in AD models, and encourage vascularization after hypoxic retinopathy.^87^

Different strategies have been investigated to facilitate BMDM infiltration into the retina. For example, insulin‐like growth factor 1 can break down the blood‐retina barrier and increase the infiltration of BMDMs into the retina.^88^ Interestingly, intravitreally injected bone marrow cells can also engraft into the retina and transform into microglia‐like cells or BMDMs.^87^ All of these findings indicate that BMDMs play protective roles against different types of retinal degeneration and that stimulating the infiltration of monocytes and bone marrow cells is a promising treatment strategy for these hard‐to‐treat diseases.

In summary, BMDMs plays complex roles in various CNS diseases. The controversial results in different studies may arise from different models, differences in disease severity in preclinical models, different pathological phases, and the accuracy of distinguishing BMDMs from microglia. Moreover, both subsets of monocytes can infiltrate into the brain in some disease conditions, such as after ischemic stroke, and these subsets of monocytes‐derived BMDMs may have distinct effects.^78^ Thus, studying these subsets separately may provide more precise treatment strategies for these diseases. It is important to note that many of these conclusions are based on the inhibition of monocyte infiltration, and caution should be taken when interpreting related results because CCR2 KO may also inhibit the process of microglial migration.^66^

## 3 BMDMS AS A VEHICLE FOR CNS GENE DELIVERY

**FIGURE 2:**
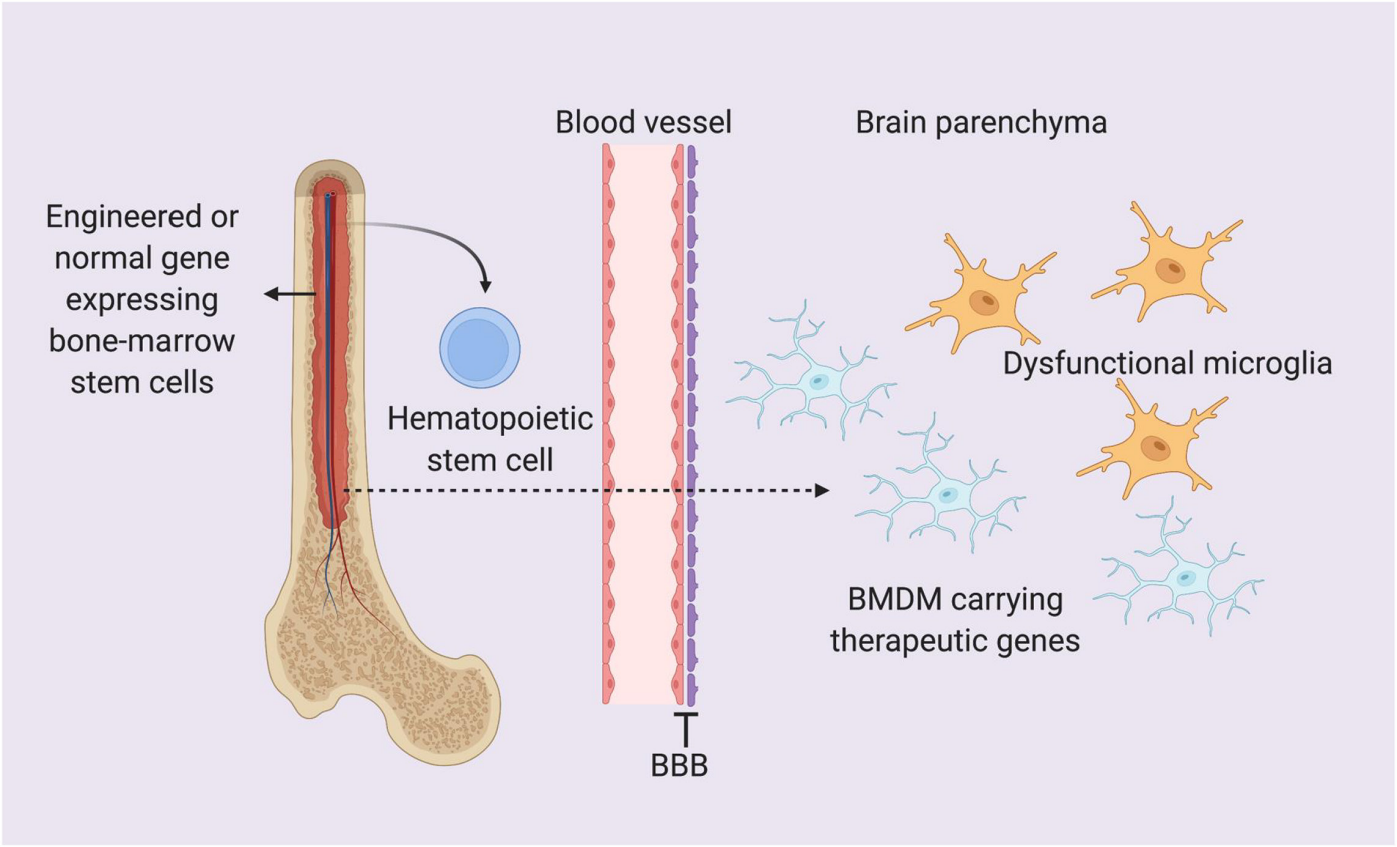
BMT delivers therapeutic genes to the brain parenchyma via BMDMs. Bone‐marrow stem cells, especially hematopoietic stem cells carrying therapeutic genes, infiltrate into the brain parenchyma via the BBB and transform into BMDMs. As a result, BMDMs express the correct genes and replace the dysfunctional microglia.

Microglia are CNS‐resident immune cells and constitute approximately 10% of the total cells in the brain.^89^ Apart from immune surveillance, microglia play vital roles in CNS development and functions, such as synaptic pruning, neurogenesis, and the prevention of excitotoxicity.^90^ Dysfunctional microglia caused by gene mutations accelerate the progression of various CNS diseases pathologies, and they can also directly cause CNS diseases, e.g., colony‐stimulating factor 1 receptor (CSF‐1R)‐related leukoencephalopathy.^91^, ^92^, ^93^, ^94^ Moreover, gene mutations in the brain are not only limited to microglia, but also occur in other cell types in some diseases, such as in lysosomal storage disorders (LSDs), and the correct or missing genes delivered by BMDM provide functional proteins to both microglia and neurons.^95^, ^96^ Therefore, introducing therapeutic genes delivered by BMDMs is a promising therapeutic strategy for CNS‐related diseases^97^, ^98^ (Figure 2).

The BBB acts as a barrier to gene therapy delivery into the CNS system, and intraparenchymal injection into specific parts of the brain is currently used in most preclinical and clinical trials. However, this is an invasive approach, and the delivery efficiency and distribution are not optimal. Moreover, multiple dosing is limited when using intraparenchymal injections. Importantly, BMDMs have been proposed as a drug delivery system for the CNS.^87^, ^99^ BMDMs can infiltrate into the brain injury sites and can widely replace resident microglia, and thus they can be used to deliver therapeutic proteins and additional nutrients to the CNS^87^, ^100^ (Figure 2).

BMDMs expressing normal genes or engineered BMDMs with therapeutic genes can be delivered by BMT. However, it is important to note that BMDMs can only graft into the brain parenchyma under special conditions following BMT. For example, whole‐body irradiation can significantly suppress the immune system and ablate the bone marrow niche, thus providing enough space for the engraftment of donor cells.^9^, ^101^ However, irradiation‐induced cell death and tissue damage may lead to other severe diseases.^102^ Another way to support BMDM engraftment is to use chemotherapies such as busulfan and cyclophosphamide prior to BMT.^8^, ^103^

Monocytes can infiltrate specifically into the injury sites under some disease conditions and then disappear upon recovery, and thus, they do not contribute to the microglial pool.^33^, ^77^, ^103^ Moreover, the numbers of BMDMs are much lower than resident microglia or dysfunctional microglia in CNS diseases models or after BMT.^104^ Therefore, increasing the microglial replacement rate by BMDMs is a critical step in delivering therapeutic genes.

BMDMs can only replace resident microglia when a microglial niche is deprived of microglia, and after microglial depletion the surviving microglia compete with the BMDMs for the empty microglial niche.^105^ One study demonstrated that microglia can be widely replaced by BMDMs after efficient microglia depletion using a CSF‐1R inhibitor.^106^ Moreover, efficient microglial depletion and BMT can achieve up to 80% microglial replacement by BMDMs.^107^ Furthermore, clinical trials of cancer therapy using CSF‐1R inhibitors have indicated that microglia can also be depleted in humans.^108^ However, large doses of these drugs are needed to achieve efficient depletion, and the safety concerns regarding extensive microglial depletion have limited their clinical use. Nonetheless, administration of a CX3CR1 inhibitor has been proposed to achieve efficient microglial replacement by BMDMs, even with only partial microglia depletion.^109^ In summary, combining BMT, microglia depletion, and CX3CR1 inhibition can be a strategy for achieving widespread microglial replacement and optimal gene therapy delivery into the CNS.

### 3.1 Amyotrophic lateral sclerosis (ALS)

The results from a chimeric mouse study showed that microglia with Cu/Zn superoxide dismutase (SOD1) mutations are neurotoxic and contribute to the ALS pathology.^110^ Moreover, wild‐type BMT has protective effects in the SOD1 ALS mouse model due to the significant engraftment of BMDMs in the brain.^111^ Furthermore, as gene carriers engineered bone marrow cells successfully delivered neuroprotective glutamate transporters to pathological lesions in the SOD1 ALS mouse model and restored motor functions.^112^ Many other cell therapies using different cell sources, including umbilical cord blood cells and mesenchymal stem cells, and given through various administration routes (intracerebroventricular, intraspinal, and intramuscular injections) have also shown modest beneficial effects.^113^, ^114^, ^115^, ^116^

LSDs make up a group of inherited metabolic disorders with a deficiency in lysosomal hydrolases that break down macromolecules (including proteins, lipids, carbohydrates, and nucleic acid) in the cells.^117^ In patients with LSDs, these macromolecules can accumulate and eventually become toxic to the CNS or other organs. CNS manifestations of LSDs can be ameliorated by BMDM engraftment followed by BMT with cells expressing the deficient hydrolase.^95^, ^101^ Another study indicated that the failure of BMT in patients with overt neurological symptoms may be due to the rapid turnover of residential microglia and insufficient BMDM engraftment. Thus, combining microglial depletion and BMT can be a solution to increase the engraftment of BMDMs in these conditions.^101^

Metachromatic leukodystrophy (MLD) is an LSD resulting from deficiency in the lysosomal enzyme arylsulfatase A and causes myelin degeneration in the CNS and peripheral nervous system. Currently, there is no available treatment for this disease; however, gene therapies via intraparenchymal injections of virus vectors carrying correct genes and ex vivo‐engineered cells might be viable options. However, the need for multiple doses and the invasiveness of the procedures limits the use of these methods in clinical practice. Engineered bone marrow cells can engraft into the CNS and achieve widespread distribution of BMDMs that express exogenous genes.^118^ For example, transplantation of engineered bone marrow‐expressing transduced arylsulfatase A was engrafted in the CNS, thus preventing neuropathological progression and rescuing behavioral deficiencies in a mouse model of MLD.^100^, ^119^

BMT is also widely used in other LSD disorders such as globoid cell leukodystrophy.^120^, ^121^ Glycolipid storage diseases are inherited disorders in which enzymes for the degradation of glycosphingolipids are missing, which eventually leads to CNS degeneration.^122^ Sandhoff disease is a glycolipid storage disease characterized by a lack of lysosomal β‐hexosaminidase and the subsequent accumulation of β‐hexosaminidase in the CNS. The introduction via BMT of BMDMs expressing the wild‐type gene has a neuroprotective effect in a mouse model of Sandhoff disease.^123^ Another example of glycolipid storage disease is GM1‐gangliosidosis, and BMT with engineered BMCs expressing lysosomal β‐galactosidase can rescue the pathological features of this disease.^124^ Taken together, BMT of BMDMs with corrected gene expression might serve as a powerful tool for treating LSDs.

### 3.2 Rett syndrome

Rett syndrome is a rare genetic and developmental neurological disorder caused by mutation in the *MECP2* gene (encoding a methyl‐CpG‐binding protein). Rett syndrome is frequently noted in girls and causes progressive motor neuron loss. Patients with Rett syndrome usually develop movement deficiency, loss of speech, and even breathing difficulties.^125^, ^126^ One study revealed that *MECP2* gene mutations in microglia can be neurotoxic, indicating the contribution of dysfunctional microglia in the progression of Rett syndrome.^127^ Moreover, BMDMs with normal *MECP2* gene expression delivered by BMT had neuroprotective effects and halted disease progression. Furthermore, BMT without BMDMs infiltration by shielding the head during irradiation showed no protective effect.^128^ These findings suggest that BMT with corrected gene expression is an effective treatment for Rett syndrome.

### 3.3 Trichotillomania

Trichotillomania is an obsessive–compulsive disorder that commonly affects juveniles. Apart from hair and skin damage, patients with trichotillomania usually develop psychiatric disorders, including emotional stress and social anxiety.^129^ The exact cause of trichotillomania is still not clear, but three mouse models (*Hoxb8* KO, *Sapap3* KO, and *Slitrk5* KO) with elevated grooming behaviors have been developed to study the pathophysiology of trichotillomania.^130^, ^131^, ^132^ Only microglia express detectable *Hoxb8* in the brain, and thus they are the most affected cells in the brain of the *Hoxb8* mutant mouse. Therefore, infiltration of BMDMs with regular *Hoxb8* gene expression after BMT can rescue pathological grooming behaviors, indicating a potential therapeutic effect.^133^, ^134^

### 3.4 X‐linked adrenoleukodystrophy (ALD)

ALD is a peroxisomal membrane transporter protein encoded by the *ABCD1* gene located on the X chromosome. *ABCD1* gene mutations can cause ALD with progressive demyelination, which is lethal. BMT has shown beneficial effects in these patients, and BMT with no suitable donor cells has also shown beneficial results for patients by using engineered cells.^135^, ^136^ The potential mechanisms of BMT for preventing CNS demyelination remain unclear, but the effect is likely due to the engraftment of BMDMs expressing normal ALD.^136^

## 4 CONCLUSION

Similar to microglia, BMDMs play dual roles in various CNS diseases, and in different disease conditions the evidence supporting the beneficial or detrimental effects of BMDMs are substantial and consistent (Table 1). For example, BMDMs play protective roles in the early stage of AD by phagocytosing Aβ, and in retinal degeneration by phagocytosing cell debris and resolving inflammation, while they can be harmful in MS and TBI by secreting proinflammatory cytokines. In addition, BMDMs play opposite roles in the early and late development of ischemic stroke. Therefore, promoting or inhibiting BMDMs within a suitable time window may slow disease progression and facilitate brain recovery.

**TABLE 1:** BMDMs play different roles in various central nervous system diseases

Disease model	BMDMs beneficial or detrimental	Possible mechanisms	Methods for blocking BMDMs methods
Low dose WNV model	Beneficial	Phagocytosis virus particles	CCR2 KO or monocyte depletion
Lethal WNV model	Detrimental	Proinflammatory	Blocking adhesion molecules or antibody against CCL2
Theiler's virus model	Detrimental	Secreting proinflammatory cytokines	CCR2 KO
MS	Detrimental	Initiating demyelination and secreting proinflammatory cytokines	Blocking adhesion molecules or CCR2 KO
TBI	Detrimental	Secreting proinflammatory cytokines, reactive oxygen species, and proapoptotic proteins	CCR2 KO or CCL2 KO or CCR2 selective antagonists
SCI	Beneficial	Secreting antiinflammatory cytokines and removing myeline or cellular debris	Monocyte depletion
	Detrimental	Inhibiting axon regeneration	Monocyte depletion
Early AD	Beneficial	Phagocytosing Aβ	CCR2 KO
Acute ischemic stroke	Detrimental	Proinflammatory	CCR2 KO
Late ischemic stroke	Beneficial	Antiinflammatory	CCR2 KO
Retinal degeneration	Beneficial	Phagocytosis of cell debris and Antiinflammatory	Inhibiting SDF1 or monocyte depletion

Abbreviations: AD, Alzheimer's disease; BMDMs, Bone marrow‐derived macrophages; CCL2, C‐C motif chemokine ligand 2; CCR2, C‐C motif chemokine receptor 2; MS, Multiple sclerosis; SCI, Spinal cord injury; SDF1, Stromal‐derived factor 1; TBI, Traumatic brain injury; WNV, West Nile virus.

Gene therapies are powerful therapeutic tools for genetic diseases, but their delivery into the CNS remains challenging. BMDMs have proven to be a powerful tool for delivering therapeutic genes to injury sites and throughout the CNS. However, BMT after either whole‐body irradiation or chemotherapy may cause severe side effects and thus should only be used in the clinic in the case of life‐threatening conditions. Moreover, the number of BMDMs is relatively low following BMT. However, combining BMT with microglial depletion can significantly increase the replacement rate of dysfunctional microglia, thus exerting potentially therapeutic effects on neurological diseases.

## AUTHOR CONTRIBUTIONS

K.Z. wrote the manuscript draft and prepared the figures. All other authors edited and revised the manuscript. All authors read and approved the final manuscript for publication.

## FUNDING INFORMATION

This study was supported by the National Nature Science Foundation of China (U21A20347), the Swedish Childhood Cancer Foundation (PR2018‐0082, PR2021‐0020), the Swedish Cancer Foundation (20‐1121‐PjF), and Swedish Governmental grants to scientists working in health care (ALFGBG‐965197).

## CONFLICT OF INTEREST

The authors declare no competing interest.

## Data Availability

Data sharing is not applicable to this article as no new data were created or analyzed in this study.
